# Immunogenicity and protective efficacy of tuzin protein as a vaccine candidate in *Leishmania donovani*-infected BALB/c mice

**DOI:** 10.3389/fimmu.2023.1294397

**Published:** 2024-01-11

**Authors:** Moodu Devender, Prince Sebastian, Vijay Kumar Maurya, Krishan Kumar, Anjali Anand, Madhulika Namdeo, Radheshyam Maurya

**Affiliations:** Department of Animal Biology, School of Life Sciences, University of Hyderabad, Hyderabad, India

**Keywords:** Leishmaniasis, Tuzin, vaccine, cytokine, antibodies, immunogenicity, antigenic

## Abstract

Visceral leishmaniasis (VL) is referred to as the most severe and fatal type of leishmaniasis basically caused by *Leishmania donovani* and *L. infantum*. The most effective method for preventing the spread of the disease is vaccination. Till today, there is no promising licensed vaccination for human VL. Hence, investigation for vaccines is necessary to enrich the therapeutic repertoire against leishmaniasis. Tuzin is a rare trans-membrane protein that has been reported in *Trypanosoma cruzi* with unknown function. However, tuzin is not characterized in *Leishmania* parasites. In this study, we for the first time demonstrated that tuzin protein was expressed in both stages (promastigote and amastigote) of *L. donovani* parasites. *In-silico* studies revealed that tuzin has potent antigenic properties. Therefore, we analyzed the immunogenicity of tuzin protein and immune response in BALB/c mice challenged with the *L. donovani* parasite. We observed that tuzin-vaccinated mice have significantly reduced parasite burden in the spleen and liver compared with the control. The number of granulomas in the liver was also significantly decreased compared with the control groups. We further measured the IgG2a antibody level, a marker of Th1 immune response in VL, which was significantly higher in the serum of immunized mice when compared with the control. Splenocytes stimulated with soluble *Leishmania* antigen (SLA) displayed a significant increase in NO and ROS levels compared with the control groups. Tuzin-immunized and parasite-challenged mice exhibit a notable rise in the IFN-γ/IL-10 ratio by significantly suppressing IL-10 expression level, an immunosuppressive cytokine that inhibits leishmanicidal immune function and encourages disease progression. In conclusion, tuzin immunizations substantially increase the protective immune response in *L. donovani*-challenged mice groups compared with control.

## Introduction

Leishmaniasis is a parasitic disease caused by various species of the protozoan parasite *Leishmania*, which belongs to the family Kinetoplastida. It is transmitted to humans through the bites of infected female sandflies from the genera Phlebotomine or Lutzomyia. The disease poses a significant threat to public health, particularly in underdeveloped nations, and is considered the second most severe parasitic illness recognized by the World Health Organization (WHO). Visceral leishmaniasis (VL) is the most severe form of leishmaniasis, which is 100% fatal if left untreated. VL is caused by *L. donovani*, *L. infantum*, and *L. chagasi* (1, 2). Thus, chemotherapy is the only option to treat leishmaniasis, but it has several limitations, such as toxicity and prolonged and expensive treatment. Vaccination is considered the most effective and definite approach for blocking the transmission and for the control of the disease. However, no promising vaccine is available against leishmaniasis for systematic use to control the disease.

Many recombinant immunogenic target-based vaccine formulations have been tested to protect against leishmaniasis at more or less advanced stages, such as LACK, A2, GP63, TSA, KMP-11, LiHyS, and LiHyp1, and a summary is provided in **Table 1** (3–11). Mice immunized with DNA plasmid (DNA LiHyP) or recombinant protein (rLiHyP) were shown to have reduced parasitism in the liver, spleen, bone marrow, and draining lymph nodes, which is associated with high levels of IFN-γ, IL-12, GM-CSF, and specific IgG2a antibodies (11). *Leishmania* amastigote-specific hypothetical protein (rLiHyJ and rLiHyG) plus saponin as an adjuvant or DNA vaccine induced higher IFN-γ production, mainly by CD8^+^ T cells, while rLiHyJ/saponin stimulated the production of this cytokine, mainly by CD4^+^ T cells, and reduced the parasite load when compared with the controls (12, 13). Mice immunized with plasmid DNA containing cysteine protease B (cpb) from *L. martiniquensis* showed significantly decreased parasite burden in the livers and spleens and increased levels of IgG2a and IFN-γ, while the IL-10 level was significantly reduced (Aunguldee et al., 2021). Mice immunized with the *L. donovani* protein disulfide isomerase-DNA construct were found to have induced Th1- and Th17-dependent immune response and T-cell proliferation (14). However, vaccination with plasmid DNA encoding KMPII, TRYP, LACK, and GP63 does not protect dogs against *L. infantum* during the experimental challenge (15). Leishmaniolysin, also known as gp63 protein, was the first recombinant antigen used in leishmaniasis vaccinations. It is a membrane protease found in promastigotes of all species of *Leishmania* parasites. The gp63 protein lacking mutant parasites has been demonstrated to be avirulent (16). The protein gp63 from *Leishmania* is a potent T-cell activator in humans, and both the native and recombinant forms of gp63 can elicit similar T-cell responses in patients with active or cured leishmaniasis. However, the recombinant form of gp63 effectively elicited T-cell responses from infected patients. Nevertheless, when using recombinant gp63 for *in-vitro* sensitization, it did not trigger T-cell responses in uninfected donors. However, T-cell responses directed against gp63 exhibit variability in human and animal models (17). Parasite surface antigen 2 (PSA-2) is the second recombinant glycoprotein (gp46/M2) of the *Leishmania* parasite and shows protection against *L. major* and *L. mexicana* infection by inducing the CD4^+^ T-cell-dependent IFN-γ production (18, 19). Lipophosphoglycan-3 (LPG3) was characterized as a heparin-binding protein and considered an important parasite virulence factor that affects the mechanisms of adhesion, internalization, and survival of the parasite during the infection (20). Lipophosphoglycan-3 recombinant protein vaccine controls hepatic parasitism and prevents tissue damage in mice infected by *Leishmania infantum chagasi* (13). Parasite-specific epitopes, leishmanial eukaryotic ribosomal protein (LeIF), efficiently generate Th1-type cytokine response in humans (21). *Leishmania*-activated C kinase (LACK) has been demonstrated to protect mice from infection when coupled with IL-12 as an adjuvant (8). A recombinant immunogenic chimeric protein from *Leishmania* amastigote LiHyp1, LiHyV, LiHyC, and LiHyG induced a specific Th1-type immune response, with significantly high levels of IFN-, IL-2, IL-12, TNF-α, and GM-CSF cytokines produced by CD4^+^ and CD8^+^ T-cell subtypes and low production of anti-leishmanial IL-4 and IL-10 cytokines (22–24). However, protection always depends on the localization and processing of parasite protein by host immune cells. Membrane proteins are still fulfilling a better role as successful vaccine candidates in many other diseases. Leishmania amastigote 2 (A2) antigen is an amastigote-specific membrane protein found in some *Leishmania* species, which was shown to be protective against VL in murine and canine models (25, 26).

**Table 1:** Types of antigenic protein tested as vaccine candidates against Leishmaniasis.

Antigen	Clinical form	Response	Outcomes	Ref.
Gp63	VL	Protection	Liver and spleen showed 86% and 81% parasite reduction. High IFN-γ, IgG2a levels persisted. Minimal IL-4	(3)
TSA	CL/L. major	Protection	The rTSA-IL12 vaccine only triggers human PBMC proliferation in those who have received protection.	(4)
rKMP-11	CL/L. major	Partial protection	Increased expression of IFN-γ, IL-4, and IL-13 was observed, whereas IL-10 expression remained unchanged.	(5)
rLeish-111f	VL	Protection	Mice reduced parasites by 91.7%, boosted IFN-γ, IL-2, TNF-β, while hamsters had similar effects.	(6)
FML	VL	Protection	85.5% reduction in liver parasite burden, 80% increase in antibody response	(7)
LACK, A2	CL, MCL, VL	Protection	Trigger a robust immune reaction	(8, 9)
LiHyP1 and LiHyP2	VL	Protection	BALB/c mice were shielded from *L. infantum* infection via Th1-type immunity induction.	(10, 11)

Tuzin is a conserved trans-membrane protein found in *Trypanosoma cruzi* with an unknown function (27). Interestingly, the tuzin gene is often adjacent to the δ amastin gene. Amastin is an abundant transmembrane glycoprotein present on the cell surfaces of parasites and essential to cell function following infection (28). *Leishmania* carries many copies of the tuzin genes spread across many chromosomes, and its persistent linkage throughout the diversification of the δ-amastin gene suggests that there is a strong functional relationship between these two gene families (29). Furthermore, tuzin is constantly associated with the same chromosomal locus with amastin genes in *Leishmania* species. The tuzin gene is found in *L. donovani*, *L. braziliensis*, and *L. infantum* but is considered a pseudogene in *L. major* and *L. tropica* (30). A study showed that DNA-based tuzin immunization protected BALB/c mice when challenged with *L. donovani* parasites, and protective immunity was correlated with higher levels of IFN-γ and IL-12 production than IL-4 and IL-10 cytokines (31). The expression of virulence genes varies depending on species-specific genes, gene polymorphisms, pseudogenes, and their localization in the organism, and all of these can affect disease pathophysiology. Hence, our aim was to investigate the function of the tuzin protein during *L. donovani* infection, and its potential as a vaccine candidate due to its specificity is confined to *Leishmania* and *Trypanosoma* parasites.

## Materials and methods

### Parasite culture

The culture of promastigotes of *L. donovani* (MH0M/IN/80/DD8) was procured from the ATCC (American Type Culture Collection), United States. Parasite culture was maintained in a BOD incubator at 25°C in M-199 medium (pH 7.4) supplemented with 15% heat-inactivated FBS, 4 mM of NaHCO_3_, and penicillin (100 U/mL) and streptomycin (100 mg/mL).

### Animals and ethical approval

All animal experiments were approved by the Institutional Animal Ethics Committee (IAEC), University of Hyderabad (UH/IAEC/RM/2020-21/16) and were maintained as prescribed by the guidelines of CPCSEA during the studies. We procured 6–8-week-old female BALB/c mice from Sainath Agencies, Hyderabad and kept them in the animal facility of the school. The mice were maintained at room temperature with a natural light cycle and fed standard rodent food and water.

### Cloning, expression, and purification of recombinant tuzin protein

The tuzin gene (LdBPK_080750) was amplified from genomic DNA using Phusion High-Fidelity DNA polymerase by PCR in Veriti 96-well thermal cycler (Applied Biosystems, Foster City, CA, USA). Briefly, the tuzin gene-specific primers for forward (5′-GCCGGATCCATGATTCCTGGAGTCGTC-3′) and reverse (5′-ATAAAGCTTTACGCCCGCCGCGGCCT-3′) were designed by inserting the *Hin*dIII and *Bam*HI restriction sites at their 5 end, respectively. The reaction program was carried out as follows: initial denaturation at 98°C for 1 min and 35 cycles of denaturation at 95°C for 30 s, annealing at 64°C for 45 s, and extension at 72°C for 1 min. The final extension was carried out at 72°C for 5 min. The amplified PCR product was purified using a QIAGEN Gel extraction kit as per the manufacturer’s instructions. The purified PCR amplicon and pET28a vector were double digested with *Hin*dIII and *Bam*HI, and the digested tuzin insert and pET28a vector were mixed in a 3:1 molar ratio in the presence of T4 DNA ligase. The ligation was carried out at 22°C overnight and transformed into *Escherichia coli* DH5a cells. To express the recombinant tuzin protein, the positive clone was transformed into *E. coli* Rosetta (DE3) strain and induced the expression of protein at 25°C overnight using 0.5 mM IPTG. The protein expression was confirmed by Western blot using an anti-His antibody.

### Antibody generation

The recombinant tuzin protein was purified using a Ni-NTA affinity chromatography column under denaturing conditions. Briefly, 500 mL of IPTG-induced culture was pelleted down at 3,500×*g* for 10 min, and the pellet was washed two times with 1× PBS. Next, the pellet was resuspended in a guanidinium lysis buffer (6 M of guanidine HCl, 20 mM of sodium phosphate pH 7.8, and 500 mM of NaCl), gently agitated for 15 min at room temperature (RT), sonicated on ice, and centrifuged for 15 min at 4,500×*g*. The supernatant was used for protein purification using a Ni-NTA purification column. After washing the column, the protein was eluted using denaturing elution buffer (500 mM of NaCl, 8 M of urea, and 20 mM of sodium phosphate, pH 4.0). The eluted protein was dialyzed for 6 h at 4°C to remove the urea using dialysis buffer [150 mM of NaCl, 10 mM of Tris (pH 8.0), and 0.1% Triton X-100]. The purified tuzin protein was used to raise the primary antibody in the rabbit as per described protocol previously. In order to generate a primary antibody against tuzin, 40 μg of the recombinant tuzin protein was combined with Freund’s complete adjuvant in equal amounts and vigorously mixed to create an emulsion. The resulting emulsion was then administered to a rabbit through subcutaneous injection. The rabbit was later injected with the first and second booster doses prepared similarly with incomplete Freund’s adjuvant, and the whole blood was drawn on the 30th day. The serum was isolated and preserved at −80°C for future use (32).

### Prediction of transmembrane and antigenicity of tuzin protein: *in-silico* study

The PSIPRED Workbench provides a range of protein structure prediction methods (bioinf.cs.ucl.ac.uk). It can predict the secondary structure of the protein, including regions of disorder and transmembrane helix packing, contact analysis, fold recognition, structure modeling, and domains and function. We used the PSIPRED Workbench software to predict the structure of the tuzin protein. The IMED (http://imed.med.ucm.es/Tools/antigenic.pl) program predicts those segments from within a protein sequence that are likely to be antigenic by eliciting an antibody response (33).

### Confirmation of tuzin protein expression in the parasite

Briefly, the parasite cell lysate was prepared from both the promastigotes and amastigotes of *L. donovani* parasites as described previously (34). The whole parasite cell lysate was resolved in 12% SDS-PAGE and transferred onto a nitrocellulose membrane using a Bio-Rad Trans-blot SD semidry transfer cell. The membrane was treated with a blocking buffer containing 5% skimmed milk solution in TBS (150 mM of NaCl, 20 mM of Tris, pH 8.0) for 1 h. Next, the membrane was incubated overnight with tuzin primary antibody raised in rabbit diluted (1:200) in a blocking buffer. Following this, the membrane was washed thrice using a TBS-Tween buffer (20 mM of Tris, 150 mM of NaCl, and 0.1% w/v Tween 20) and then exposed to a secondary antibody anti-rabbit conjugated with HRP (Cat log: 31460, Invitrogen, Carlsbad, USA) for 1 h at RT. The TBST buffer was used to rewash the membrane, and the respective protein bands were visualized using the FemtoLUCENT™ PLUS-HRP Chemiluminescence Detection System and the ChemiDoc system (Bio-Rad, California, USA).

### Immunofluorescence assay to localize the tuzin protein in the parasite

Briefly, *Leishmania* parasites were fixed in 4% formaldehyde in PBS for 20 min at RT. The fixed parasites were then washed three times with 1× PBS and allowed to attach to glass coverslips and air-dried. Once air-dried, they were treated with ice-cold methanol (−20°C) for 5 min and then blocked with 1% BSA in PBS for 30 min. Afterward, an anti-tuzin primary antibody was added in a 1:200 dilution and incubated for 1 h and rewashed three times with PBS with 0.1% Tween 20. Coverslips were subjected to a secondary antibody (goat anti-rabbit IgG antibody with FITC) in a 1:300 dilution. Once again, it was washed three times with PBS 0.1% Tween 20, and finally, the coverslip was fixed onto glass slides using Vectashield, a mounting medium that includes DAPI. Finally, the slides were examined under a confocal microscope to detect fluorescence (34).

### Tuzin immunization and parasite-challenged BALB/c mice

A total of 25 mice were randomly assigned into five groups, each containing five mice: group 1: healthy control, group 2: healthy + adjuvant (Freund’s adjuvant), group 3: infected control, group 4: adjuvant-immunized (Freund’s adjuvant) + challenged with *L. donovani* parasites, and group 5: tuzin-immunized (tuzin + Freund’s adjuvant) + challenged with *L. donovani* parasites. Forty micrograms of tuzin protein and 50 µL of adjuvant were used to immunize the BLAB/c mice through a subcutaneous route. Two booster doses were given with a 2-week gap between each administration. Mice were exposed to *L. donovani* parasites in the form of metacyclic promastigotes 4 weeks after receiving the last booster dosage. In brief, the metacyclic promastigote parasite was isolated by subjecting a stationary promastigote culture to Ficoll gradient centrifugation. The parasites were washed with PBS and counted, and subsequently, 1 × 10^7^ parasites were injected into each mouse through the tail vein. After 8 weeks of the challenge, the mice were euthanized, and peripheral blood and visceral organs such as the spleen and liver were collected for subsequent studies. All animal studies were conducted twice and obtained consistent results. The data shown here are a representative dataset from one of the independent animal studies.

### Determination of parasite burden in BALB/c mice

#### Microscopic method

Briefly, touch biopsy was performed on a glass slide using a mouse spleen, and then the slide was allowed to dry. Thereafter, the slides were fixed in methanol for 5 min and then stained for 30 min using Giemsa stain (HiMedia, Maharashtra, India). Then, the slides were washed gently and air-dried. The slides were visualized under a light microscope to count the parasites. For all infected and tuzin-immunized groups, the intracellular amastigote parasites were counted in 100 macrophages (35).

### Real-time PCR

#### DNA extraction

One hundred microliters of homogenized liver tissue was lysed in 80 μL of lysis buffer along with 20 μL of proteinase K. Then, the mixture was incubated overnight at 56°C to ensure full lysis. Next, the QiAmp DNA Mini Kit (Qiagen, Hilden, Germany) was used to isolate the DNA as described by the manufacturer.

#### Standard curve

A standard curve was made to determine parasite load by estimating *Leishmania* DNA through serial dilutions. The QIAamp DNA mini kit was used to extract the DNA of *L. donovani* from 1 × 10^8^ promastigotes, quantified, and considered as a stock DNA solution. The stock DNA was diluted at a concentration assumed to be 10^6^ parasites/mL (as 120 *Leishmania* parasites contain 10 pg of DNA). The stock DNA solution was subjected to 10-fold serial dilutions, resulting in eight points on a curve that spans 0.1–10^6^ parasites (36).

#### qPCR experiment

SYBR Green-based qPCR was used to quantify the parasite’s kinetoplast DNA (kDNA) in the liver for accurate sensitivity. Twenty microliters of PCR reaction consisted of SYBR Green master mixture (2×) from TaKaRa (Shiga, Japan), Milli-Q water, DNA template, and forward and reverse primers of kDNA. The initial qPCR setup consisted of an initial incubation for 2 min at 50°C followed by denaturation for 10 min at 95°C, 40 cycles for 15 s at 95°C, and 1 min at 60°C for each. The ABI SDS software was used to create standard curves, mean values, and amplification plots, and the melting temperatures of each amplicon were calculated using the same software. Negative controls, including a healthy control and no template controls, were included in each plate to address contamination concerns (37).

### Quantitation of serum IgG1 and IgG2a

The level of IgG1 and IgG2a antibodies in mouse serum of different experimental groups was measured using the standard indirect ELISA method. The recombinant tuzin protein 100 ng/well was coated in 96-well ELISA plates and incubated overnight at 4°C. Subsequently, the plate was washed and blocked with 1% BSA at room temperature for a duration of 2 h. Next, the plate was incubated with diluted serum of all groups (1:100) and kept at 37°C for 1 h. Following this, the plate was washed with PBS-Tween 20. Next, HRP-conjugated rat anti-mouse IgG1 and IgG2a antibodies were added (dilution 1:10,000, Abcam^®^, Waltham, USA) to each well and incubated for 1 h at 37°C. Once again, the plate was washed thoroughly and then added with 100 µL of a substrate consisting of 1× TMB/H_2_O_2_ and further incubated for 30 min at room temperature in the dark. The reaction was stopped by adding 50 μL of stop solution (1 N of H_2_SO_4_), and the color intensity of the plate was measured at 450 nm in a microplate reader (LTEK, South Korea) (38).

### Histopathological studies

Liver tissue sections were collected from all mice groups and fixed in 10% formalin saline buffer. These tissue samples were sent to the LV Prasad Eye Institute in Hyderabad for hematoxylin–eosin (H&E) staining. The number of granulomas per 50 fields was counted at ×40 using a light microscope (Leica, Wetzlar, Germany) (39).

### Nitric oxide quantification

The splenocytes from all mouse groups were grown in 12-well culture plates containing a complete RPMI medium. The cells were incubated with and without 80 µg/well of soluble *Leishmania* antigen (SLA) for 72 h at 37°C in a CO_2_ incubator. The resulting supernatant was collected and examined for nitric oxide (NO) levels. Briefly, 100 µL of the culture supernatant was added to a 96-well ELISA plate and then treated with 100 µL of Griess reagent (Sigma, Sigma-Aldrich, Burlington, USA). The plate was then incubated for 30 min at RT. The NO was measured at 540 nm, and the nitrite levels were determined using standard curves produced from various concentrations of NaNO_2_ (10–100 μM) (40).

### Reactive oxygen species analysis

Intracellular reactive oxygen species (ROS) levels were measured using a cell-permeable dye known as H_2_DCFDA (2,7-dichlorodihydrofluorescein-diacetate). ROS was measured in splenocytes from various groups of mice. Briefly, splenocytes (2 × 10^6^ cells/well) were treated with or without SLA (80 µg/well) and incubated for 3 days in a CO_2_ incubator at 37°C. The supernatant was collected, and the cell pellet was washed with PBS and subsequently incubated with 10 µM of H_2_DCFDA in PBS in the dark for 30 min at RT. The green fluorescence of the H_2_DCFDA reaction with ROS was measured in the form of mean fluorescence intensity (MFI) by flow cytometry (BD LSR Fortessa™ instrument and FACSDiva software) (41).

### Cytokine gene expression analysis

#### RNA isolation and cDNA preparation

Following the manufacturer’s instructions, RNA was extracted from splenocytes in all mouse experimental groups using the QIAGEN RNeasy mini kit. After that, 1 µg of total RNA was used to synthesize cDNA using the TaKaRa PrimeScript 1st Strand DNA synthesis kit. The resulting amplified cDNA was stored at −80°C for further use.

#### Real-time quantitative PCR

Briefly, cDNA was used as a template to amplify the target genes using their respective primers (**Table 2**) and SYBR Premix Ex Taq (2X) from TaKaRa. The qPCR reaction was prepared as per the kit instructions. qPCR was set up for 40 cycles; initial 2 min at 95°C, followed by denaturation for 15 s at 95°C, and annealing for 30 s at 60°C. The qPCR results were analyzed by StepOnePlus™ software of Applied Biosystems (Waltham, USA). The relative fold expression of the target gene was measured using the 2^−ΔΔct^ technique. The mean ± SD from two independent experiments was used to calculate the relative expression (42).

**Table 2:** Mouse cytokine gene-specific primers were used for gene expression analysis in the spleen of mice.

Primer		Sequence (5′–3′)
IFN-ϒ	FP	TCAAGTGGATAGATGTGGAAGAA
	RP	TGGCTCTGCAGGATTTTCATG
IL-10	FP	GGTTGCCAAGCCTTATCGGA
	RP	ACCTGCTCCACTGCCTTGCT
IL-4	FP	ACAGGAGAAGGGACGCCAT
	RP	GAAGCCCTACAGACGAGCTCA
TGF-β	FP	TGACGTCACTGGAGTTGTACGG
	RP	GGTTCATGTCATGGATGGTGC
GAPDH	FP	CAAGGCTGTGGGCAAGGTCA
	RP	AGGTGGAAGAGTGGGAGTTGCTG

#### Quantitative cytokine ELISA

Briefly, plasma samples were prepared by centrifuging the blood containing EDTA at 3,500 rpm at 4°C for 10 min. The levels of IFN-γ and IL-10 cytokines in the plasma were measured according to the ELISA kit instructions (FineTest, Wuhan Fine Biotech Co., Ltd., Wuhan, China). The range and sensitivity limit for IFN-γ are 31.25 to 2,000 pg/mL and 18.75 pg/mL, respectively, while the range and sensitivity limit for IL-10 are 15.625 to 1,000 pg/mL and 9.375 pg/mL, respectively. Briefly, 100 µL of the diluted serum (1:2) was added to the appropriate wells of the ELISA array plate, which included all specific cytokine capture antibodies. After 90 min of incubation at RT, the plate was washed. Then, 100 µL of the detection biotin-labeled antibody was added to the appropriate wells and incubated for 1 h at RT. Subsequently, 100 μL of diluted HRP-streptavidin conjugate was added to all wells and incubated at RT for 30 min. After washing, 90 μL of the TMB substrate solution was added to each well and incubated for 20 min at RT in the dark. Finally, 50 μL of stop solution was added to each well, and the absorbance of the products was measured at 450 nm in a microplate spectrophotometer (INNO, LTEK, Korea). The standard graph was plotted for IFN-γ using (*y* = 0.0008*x*) with a correlation coefficient (0.9878) and for IL-10 (*y* = 0.0009*x*) with a correlation coefficient (0.9931). The target concentrations of the sample (IFN-γ and IL-10) were interpolated from their respective standard curves.

#### Statistics

Data analysis was performed on GraphPad Prism 7, and statistical significance was determined at a significance level of *P <*0.05. The *t*-test was employed to calculate statistical significance when comparing groups individually.

## Results

### Tuzin is constitutively expressed in both stages of the *Leishmania donovani* parasite

**Figure 1:**
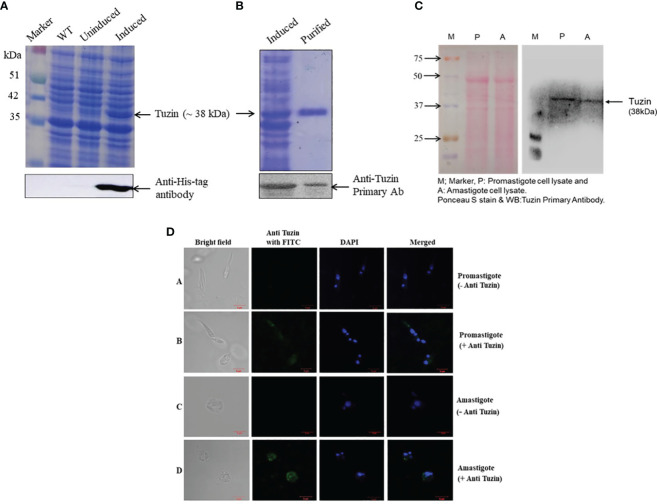
Expression, purification, and confirmation of tuzin protein in *Leishmania donovani* parasites: **(A)** SDS-PAGE; expression of recombinant tuzin protein in *Escherichia coli*. Rosetta (DE3) strain and confirmation of expression by Western blot using anti-His tag antibody. **(B)** SDS-PAGE of purified recombinant tuzin protein and confirmation of tuzin protein by Western blot using anti-tuzin primary antibody raised in rabbit. **(C)** Confirmation of tuzin protein expression in *L. donovani* parasites after probing with anti-tuzin antibody by western blot. **(D)** Tuzin protein localization by immunofluorescence assay: panel 1—bright field; panel 2—anti-tuzin probed with FITC fluorescence; panel 3—DAPI fluorescence; panel 4—merged. **(A, B)** Promastigote control (without anti-tuzin antibody) and test (with anti-tuzin antibody), respectively; **(C, D)** amastigote control and test, respectively.

The tuzin gene was cloned into a pET28a vector having both N- and C-terminal His6-tag. Then, the recombinant plasmids were transformed into *E. coli* Rosetta (DE3) cells for expression studies. The expression of recombinant protein was induced with IPTG and confirmed by Western blot using an anti-His tag antibody (**Figure 1A**). Then, the recombinant protein was purified under denaturing conditions, and the primary antibody was raised against purified tuzin protein in rabbits. Furthermore, the specificity of the raised antibody was confirmed with purified recombinant protein (**Figure 1B**). Thereafter, to assess the expression of tuzin protein in *L. donovani* parasites, we probed the primary antibodies with cell lysate of promastigote and axenic amastigote parasites (**Figure 1C**), and the Western blot results suggest that tuzin is constitutively expressed in both stages of the *L. donovani* parasites. The confocal microscopy results revealed that the tuzin protein was probably localized at the plasma membrane of the parasite (**Figure 1D**). Our results clearly demonstrated that the tuzin protein is constitutively expressed in *L. donovani* parasites.

### Tuzin is a transmembrane protein and is antigenic in nature

**Figure 2:**
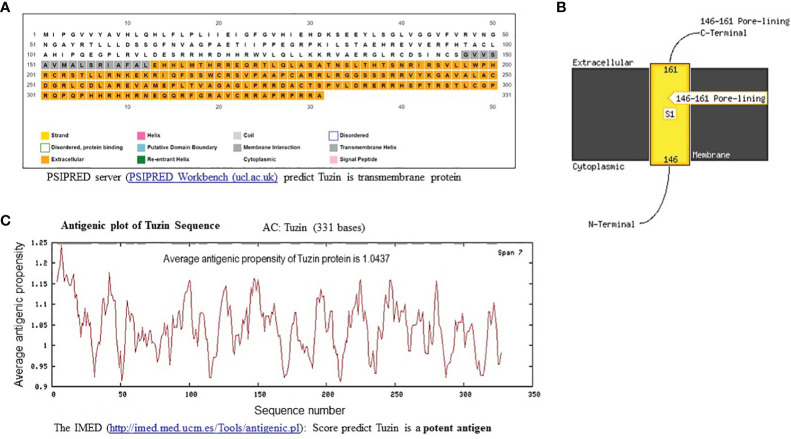
*In-silico* structural and antigenic analysis of tuzin protein. **(A)** PSIPRED-based transmembrane prediction report for the tuzin protein shows the color coding of the sequence nature of the tuzin protein: the gray shading indicates the transmembrane helix, the colorless portion represented the cytoplasmic portion, and the orange color indicates the extracellular helix sequence of the tuzin protein. **(B)** The figure illustrates the detailed visualization of the N-terminal cytosolic helix and C-terminal extracellular helix. **(C)** The antigenicity propensity scores are generated by IMED prediction; the average antigenicity propensity score ≥1 is considered a potent antigen. The average antigenicity propensity score for the tuzin protein is 1.0437, considered a potent antigen.

The PSIPRED Workbench platform was used to predict the structure of the tuzin protein. PSIPRED is a simple and accurate secondary structure prediction method, incorporating two feed-forward neural networks performing an analysis on the output obtained from PSI-BLAST. The results showed that the tuzin protein is a transmembrane protein containing N-terminal cytosolic and C-terminal extracellular helix (**Figures 2A, B**). Furthermore, we used the IMED program to predict antigenic segments within a tuzin protein sequence that are likely to be antigenic by eliciting an antibody response. An IMED score of more than 0.7 is considered to be a potent antigen. The results showed that tuzin protein antigenic propensity is 1.0437, suggesting that the tuzin protein could contain a potent antigenic domain and can be considered a vaccine candidate (**Figure 2C**).

### Tuzin vaccination efficiently controls parasite burden in the spleen of mice

**Figure 3:**
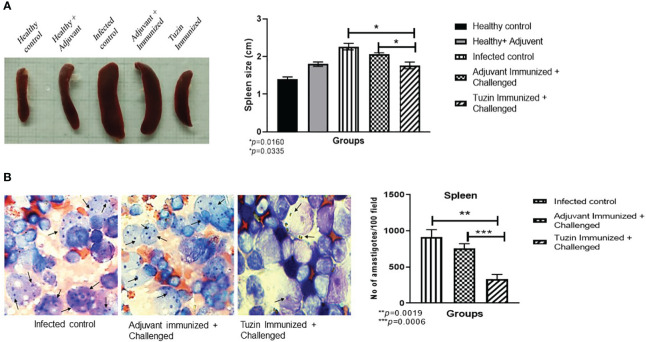
Measurement of spleen size and parasite load. **(A)** Spleens from all the experimental groups of mice were collected and measured using a scale. **(B)** Intracellular amastigotes were counted in the spleen using Giemsa staining: the graph shows the amastigote numbers for 100 fields. The values showed significant differences between the infected control and the tuzin-immunized group and were calculated using Student’s *t*-test (***P < 0.05). The data shown here are a representative dataset from one of the independent animal studies. *p ≤ 0.05, **p ≤ 0.005.

Metacyclic promastigotes that are administered intravenously may directly influence the visceral organs by infecting tissue macrophages and dendritic cells (DCs), which would then disseminate the infection by obliterating the host immune system (43). In visceral leishmaniasis, splenomegaly and hepatomegaly are the hallmarks of the disease. Hence, we measured the size of the spleen across all groups of mice. We observed that the spleen of tuzin-immunized mice was notably smaller (*P* < 0.0160) when compared with the infected control group (**Figure 3A**), suggesting that tuzin vaccination reduced the splenomegaly which could be due to the lower parasite burden. The parasite burden in the spleen was determined by light microscopy. The number of intracellular amastigote parasites was counted for 100 nucleated cells of each mouse, and we found that the infected control mice had a significantly higher parasite load in comparison to mice immunized with tuzin protein (*P* < 0.0019) (**Figure 3B**).

### Tuzin induces a hepatic granulomatous response and decreases the parasite burden in the liver

**Figure 4:**
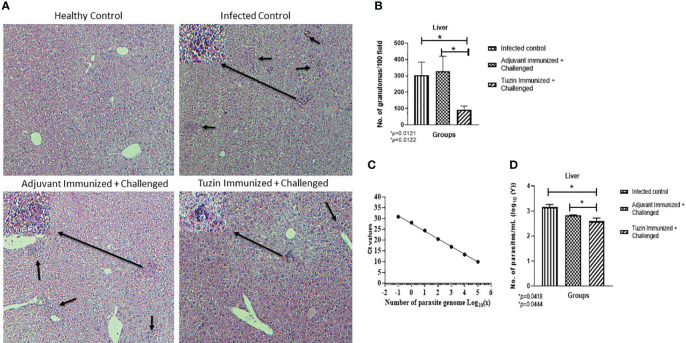
Histological analysis of liver tissue of various groups of mice 8 weeks after infection/challenged. **(A)** Healthy control, infected control, adjuvant-immunized + challenged, and tuzin-immunized + challenged groups. **(B)** Granuloma counts; infected control, adjuvant-immunized + challenged, and tuzin-immunized + challenged group. **(C)** Standard curve plotted with various dilutions of genomic DNA of *L. donovani* parasites. **(D)** Parasite load in liver tissue quantified by qRT-PCR. The significant difference between the infected control and the tuzin-immunized + challenged group was calculated using Student’s *t*-test (*P < 0.05).

Rapid granuloma formation accelerates the parasite killing in the liver which can be infiltrated by IFN-γ + T cells and NO-producing macrophages in response to the Kupffer cells that produced chemokines and myeloid DC-derived IL-12 (44). Histological analysis of the liver tissue of different groups of mice confirmed that the size and integrity of granulomas were comparably smaller in the tuzin-immunized parasite-challenged group compared with the infected control group (**Figure 4A**). The tuzin-immunized parasite-challenged group showed significantly fewer granuloma formations (*P < 0.0121) than the infected control (**Figure 4B**). The number of granulomas per 50 fields was counted at ×10 using a light microscope. The parasite burdens in the liver of mice were also estimated by quantitative real-time PCR (qRT-PCR) using parasite-specific kDNA. The genomic DNA was isolated from mouse liver homogenate to estimate the parasite load, while genomic DNA isolated from the *L. donovani* parasite was used to plot a standard curve. To perform the qRT-PCR, equal amounts of DNA from different groups of mice were used as templates. The control group consisted of healthy mice. The average Ct values obtained by RT-PCR were 30.868, 17.635, 16.66, and 18.078 for the healthy + adjuvant, infected control, adjuvant-immunized + challenged, and tuzin-immunized + challenged groups, respectively. The standard curve was plotted with the various dilutions of genomic DNA of *L. donovani* (**Figure 4C**). The parasite loads were determined for each group using GraphPad Prism by calculating the linear regression of the Ct values and interpolating the Ct value of the infected control, adjuvant-infected control, and tuzin-immunized groups in the *x* value of the standard curve. The results of the qRT-PCR analysis indicated a significant reduction in parasite load in the tuzin-challenged group compared with the infected control group (*P < 0.0418) with a 95% confidence interval (**Figure 4D**). The results showed that tuzin-immunized mice cleared the parasites more efficiently than its control, suggesting that tuzin efficiently induced the protective immune response in these mice.

### Tuzin immunization induces IgG2a antibody response

**Figure 5:**
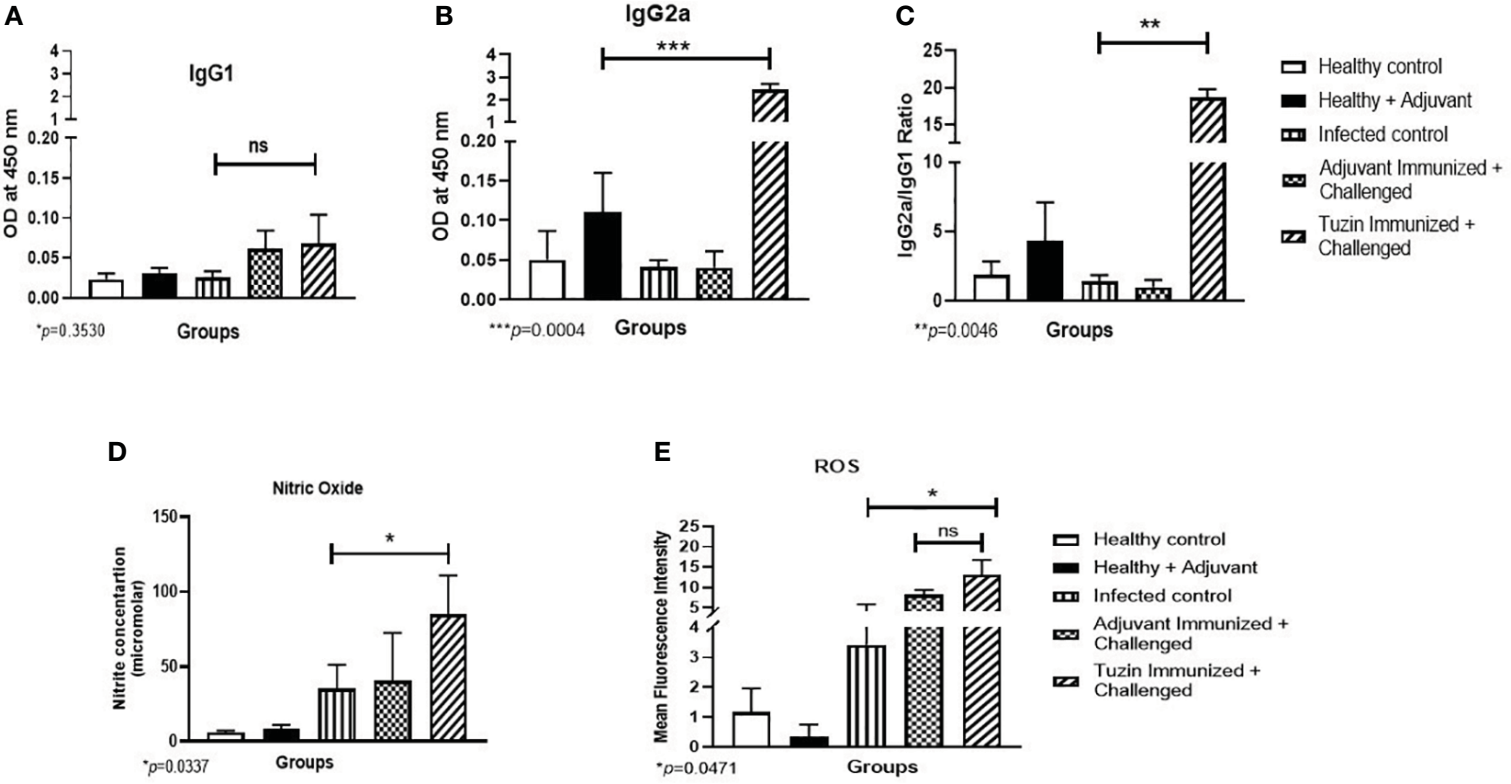
The titer of IgG1 and IgG2a antibodies measured by ELISA. The sera of all mice groups were collected after 8 weeks of infection/challenged. **(A)** Level of IgG1. **(B)** Level of IgG2a. The data are presented as the mean ± SD for each group. Significant differences between the infected control and the tuzin-immunized + challenged group (***P < 0.005) were calculated using Student’s *t*-test. **(C)** The IgG2a/IgG1 ratios were measured and calculated using Student’s *t*-test. **(D)** Nitric oxide level was measured after 8 weeks of infection/challenged; the nitrite level of the sample was calculated using standard curves made from various concentrations of NaNO_2_. **(E)** ROS was also measured after 8 weeks of infection/challenged. The green fluorescence of H_2_DCFDA was measured using flow cytometry, and mean fluorescence intensity (MFI) was represented as a bar graph. *p ≤ 0.05, **p ≤ 0.005.

Humoral response is one of the key factors in determining immunological activation during leishmaniasis, which manifests as an antibody-dependent protection. Immunoglobulin (IgG) subclass and quantity are determined by the type of CD4^+^ T helper (Th) subset response. IgG2a and IgG1 immunoglobulin classes have been shown to flip in response to IFN-γ and IL-4 cytokines, respectively (45). We analyzed the humoral immune response induced in the tuzin-immunized parasite-challenged group compared with the infected control groups. We measured the levels of *Leishmania*-specific IgG1 and IgG2a antibodies in the serum of mice 8 weeks after the challenge. We observed that the levels of IgG1 antibodies in the tuzin-immunized parasite-challenged group were comparatively unaffected compared with the control groups (**Figure 5A**). In contrast, the tuzin-immunized parasite-challenged group exhibits significantly higher IgG2a titers compared with the control (*P* < 0.004) (**Figure 5B**), indicating that tuzin potentially induced Th1-specific antibodies. The ratios of IgG2a/IgG1 antibodies were significantly higher in the tuzin-immunized challenged mice groups compared with their control (**Figure 5C**). These findings suggest that tuzin immunization skewed the immune response toward a cell-mediated immune response, mainly characterized by increased levels of IgG2a antibodies during *L. donovani* infection.

### Tuzin induces NO and ROS-mediated parasite killing

To eliminate intracellular parasites from macrophages, reactive oxygen species is one of the key molecules used by macrophages for their defense against pathogens (46). Activated macrophage generates harmful oxygen metabolites such as superoxide anion (O^−2^), NO, and hydrogen peroxide (H_2_O_2_), which are effective in killing intracellular parasites. In this study, we collected splenocyte culture supernatant to measure NO levels, and the cells were used for ROS analysis by flow cytometry. We found that the tuzin-immunized parasite-challenged group displayed a significant increase in NO production upon stimulation with SLA, compared with the infected groups (**Figure 5D**). Furthermore, ROS production was measured using H_2_DCFDA dye and analyzed by flow cytometry. The MFI showed higher ROS levels in the tuzin-immunized parasite-challenged group compared with the infected control group (**Figure 5E**). Our results indicate that a significant increase in NO and ROS in SLA-stimulated splenocytes of the tuzin-immunized group was associated with enhanced anti-leishmanial activities.

### Tuzin immunization suppresses IL-10 cytokine expression to improve the Th1 type of immune response in mice

**Figure 6:**
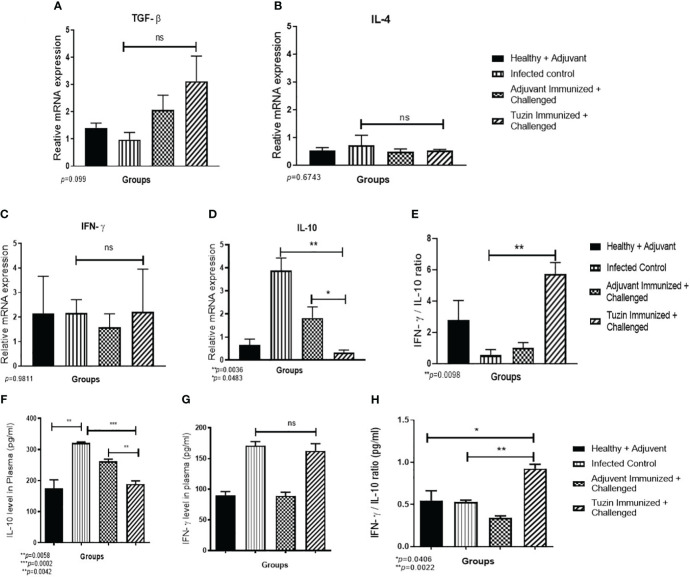
The relative gene expression analysis in the spleen of mice using RT-qPCR. RT-qPCR analysis of Th1- and Th2-specific cytokine relative expression in the spleen of mice. Healthy, infected groups, adjuvant-immunized, and tuzin-immunized + challenged groups and Th1/Th2 cytokines such as IL-4, TGF-β, and IFN-γ levels were unchanged in all groups of mice **(A–C)**, whereas IL-10 cytokine was significantly downregulated in tuzin-immunized + *L. donovani*-challenged groups **(D)**. However, the IFN-γ/IL-10 ratio was significantly high in tuzin-immunized and challenged mice compared with the other groups **(E)**. Estimation of cytokines (pg/mL) released in the serum of various groups of mice by the ELISA method. Healthy, infected control, adjuvant-immunized + challenged, and tuzin-immunized + challenged groups. There were no significant changes in IFN-γ level **(F)**; however, the most interesting point is that the IL-10 cytokine was found significantly lower in tuzin-immunized + challenged groups compared with the control **(G)**. Total IFN-γ/IL-10 ratio was also significantly higher in tuzin-immunized challenged groups compared with the control **(H)**. *p ≤ 0.05, **p ≤ 0.005, ***p ≤ 0.0005.

The pathogenesis of leishmaniasis and host defense primarily depends on two critical cytokines: IFN-γ and IL-10. The latter plays an important role in disease progression during *Leishmania* infection. Quantitative real-time PCR was performed to determine the expression levels of IFN-γ and IL-10 cytokines in different experimental groups. IL-10 decreases the antiparasitic effect of macrophage by downregulating the Th1-specific cytokine response and upregulating Th2-specific cytokines (TGF-β and IL-4), which also suppresses macrophage activation (47). In the progression of the VL, higher levels of IL-10 are more critical than a deficiency in IFN-γ. In killing the intracellular parasites, IL-10 substantially resists IFN-γ-mediated macrophage activation while somewhat inhibiting IFN-γ production. We observed the expression of the anti-inflammatory cytokines such as TGF-β and IL-4 unchanged (**Figures 6A, B**), while IL-10 was noticeably decreased in the tuzin-immunized challenged group when compared with the control group (**Figure 6C**). In contrast, the IFN-γ level remained unchanged (**Figure 6D**). The presence of IL-10 cytokine is essential for the survival of parasites within the host macrophages. The result indicates that tuzin efficiently downregulates IL-10 expression causing a protective immunity in mice. The viability of intracellular parasites during VL was also determined by measuring the IFN-γ/IL-10 ratio. The high IFN-γ/IL-10 ratio suggests that the immune response shifts toward the Th1 phenotype (48). We observed that the tuzin-immunized group showed a significant increase in IFN-γ/IL-10 ratio compared with the control (**Figure 6E**), suggesting that tuzin as a potential vaccine candidate efficiently promotes the immune response by inducing Th1 type of protective cytokine response in the tuzin-immunized *L. donovani*-challenged group.

The immune dysfunction during active VL caused by *L. donovani* has been associated with the incapability of lymphocytes to produce cytokines in response to *Leishmania*-specific antigen stimulation (49). Therefore, we measured the potential secreted cytokines IFN-γ and IL-10 in the plasma of mice by ELISA. Interestingly, we observed the same results as we have seen in the gene expression analysis of spleen cells. There is no significant difference in IFN-γ level in both infected control and tuzin-immunized challenged groups (**Figure 6F**), whereas IL-10 levels were drastically reduced in the tuzin-immunized challenged groups (**Figure 6G**). The total IFN-γ/IL-10 ratio was also significantly higher in the tuzin-immunized challenged groups compared with the control (**Figure 6H**).

## Discussion

The most cost-efficient strategy to prevent infectious diseases seems to be vaccines. Designing safe and effective vaccine regimens for clinical use remains critical. The protective immunity against *Leishmania* infection depends on increased IFN-γ and INF-α production, a marker of Th1 response (50, 51). Antigens that can trigger Th1 response could be considered promising proteins to ward off infection (52). Therefore, finding the ideal vaccine candidates and the right adjuvant is crucial for creating an effective vaccination. The membrane proteins are among the target proteins to consider as a vaccine candidate since they are present on the cell surface of the pathogen. Tuzin is a membrane protein that frequently coexists with the amastin transmembrane protein of the *Leishmania* parasites. Therefore, we initially used *in-silico* prediction tools to determine the immunogenicity of the tuzin protein, and we found that tuzin has the ability to elicit an immune response. We observed a lower parasite burden in the spleen of the tuzin-immunized group compared with the control groups, which might be associated with tuzin’s immunogenicity and capacity to incite a protective immune response against the parasites. The liver of normal mice infected with *L. donovani* produces tissue granulomas by resident macrophages, which are designated as Kupfer cells in the liver. Furthermore, it leads to the recruitment of monocytes and other immune cells and forms granulomas. The granuloma comprises a heterogeneous population of immune cells (both infected and uninfected macrophages, monocytes, and dendritic cells). Later, the expansion of granuloma is mediated by the IFN-γ-induced inflammatory monocytes, is more in number and actively involved in the phagocytosis of infected host cells, and lowers the transmission of parasites to other resident immune cells (53, 54). The immune cells recognize and respond to these granulomas by activating monocytes and T cells that release cytokines, which effectively control the dissemination of *Leishmania* parasites in the liver (55). We found that mice immunized with the tuzin antigen showed a significant reduction in parasite load in the liver compared with the control groups. Our results also showed that the tuzin-immunized group had fewer and smaller granulomas in their liver tissue compared with the infected control group. Most of the cells in a granuloma are T cells, and mature T cells in granulomas can release IFN-γ (56). Higher levels of IFN-γ were associated with increased nitric oxide production in activated macrophages promoting the suppression of intracellular parasite growth, while IL-10 was associated with *Leishmania* pathogenesis, leading to disease progression and parasite persistence (57).

We used IgG1 and IgG2 responses as indicators to evaluate tuzin immunogenicity in tuzin-immunized parasite-challenged mice since their response is entirely T-cell-dependent (58). IgG2a antibodies imply a protective Th1 immune response, whereas IgG1 antibodies indicate a Th2 immune response (59). It has been noted that *Leishmania* infection triggers host IgG1, which increases IL-10 secretion which aids in the worsening of the disease (60). Serological data showed that mice immunized with the tuzin antigen induced strong humoral responses after the parasite challenge. IgG1 antibody levels were nearly the same in all mice groups, including the immunized groups. However, serum samples from immunized mice had higher IgG2a antibody levels, suggesting that the infection was contained by inducing the protective Th1 response in the tuzin-immunized parasite-challenged groups. Heat-killed *Leishmania* parasites coupled with different adjuvants significantly increased the production of IgG2a, indicating the generation of a protective Th1 response (61). A DNA vaccine containing cysteine protease B (cpb) gene or tuzin gene-vaccinated mice showed significantly increased levels of IgG2a and IFN-γ, while the IL-10 level was significantly reduced when compared with the control group (31, 62). DNA plasmid (DNA LiHyP) or recombinant protein (rLiHyP) was also associated with high IgG2a antibodies and low IgG1 antibodies (63). Our study revealed that the tuzin protein-immunized group had significantly higher IgG2a antibody titers and lower IgG1 levels compared with the control groups, suggesting that tuzin immunization efficiently triggers IgG2a-dependent protective immune response in the immunized groups.

It is known that PBMCs from active VL patients do not produce proinflammatory cytokines such as IFN-γ and IL-12 in response to *Leishmania* antigens (43, 64, 65). However, IFN-γ response can be detected a few months after successful completion of the treatment against the disease, as well as in individuals with subclinical or asymptomatic infection (66–68). IL-10 is believed to play a key role in promoting the progression of VL by priming host macrophages to enhance parasite survival in mice (69). A study also suggests that IL-10 produced by CD4^+^ T cells is a potential autocrine inhibitor of IFN-γ production and promotes parasite persistence in visceral organs; however, blocking of IL-10 cytokine signal enhances parasite clearance in human VL (57). We observed that the infected control mice displayed the highest levels of IL-10 production when compared with tuzin-immunized mice. Tuzin immunization significantly controlled IL-10 production in challenged groups. It is known that inhibiting IL-10 in VL serum can stop the replication of *L. donovani* in macrophages and boost the IFN-γ response by antigen-stimulated PBMCs (49). The persistent stimulation of parasite antigen induces proinflammatory cytokines, which activate the expansion of CD4 T cells to produce the regulatory cytokine IL-10. The endurance strategies of the *Leishmania* parasite within the host are attributed to increased production of the regulatory cytokines TGF-β and IL-10. The elevated level of these cytokines inhibits the expression of IFN-γ and inducible NO synthase and suppresses the macrophage microbicidal functions by decreasing the NO production (70–72). To further understand the association between the immune response and the level of protection in tuzin-immunized mice, a study was conducted to evaluate the expression of IFN-γ and IL-10 as crucial cytokines in VL pathogenesis. The clinical study on VL patients demonstrates that IL-10 is the major cytokine that blunts the activity of the IFN-γ (57).

Interestingly, the tuzin-immunized and parasite-challenged groups showed a basal level of IFN-γ expression and decreased IL-10 expression. The same cytokine level was reflected in the plasma of immunized mice groups, suggesting that IFN-γ is incapable of exerting its action. At the same time, tuzin immunization strongly downregulates IL-10 expression. Even though the IFN-γ level is at the basal level, the IFN-γ/IL-10 ratio was increased in immunized mice, indicating the protective immunity of tuzin. The findings showed that the tuzin-immunized group had significantly reduced expression of IL-10 compared with the control group, while IFN-γ levels remained the same. This suggests that the tuzin protein can be potential antigens which are able to offer a protection against infection. The survival of *Leishmania* parasites during VL also depends on the ratio of IFN-γ/IL-10. If their ratio is low, then parasites are able to survive and proliferate inside the host. Conversely, when the ratio of IFN-γ/IL-10 is elevated, the immune response switches to the Th1 phenotype toward a protective immune response (48). The tuzin-immunized group showed a much higher IFN-γ/IL-10 ratio than the infected control group, indicating a Th1-type immune response which is considered protective immunity.

Unlike IL-10, TGF-β inhibits the functions of TNF-α and IFN-γ and controls the expression of inducible nitric oxide synthase (iNOS), which includes inhibition of T-cell proliferation and MΦ activation (44, 73). The elevated level of TGF-β cytokine inhibits the expression of IFN-γ and inducible NO synthase and contributes to inadequate NO production in VL (70, 74). Macrophages exposed to physiological doses of TGF-β were unable to eliminate the intracellular parasite, although the respiratory burst caused by phagocytosis remains intact (70). In our study, tuzin immunization showed a significant increment in NO, which correlates with the downregulation of IL-10 expression and maintains the TGF-β level at the basal level in tuzin-immunized mice, suggesting its protective efficacy as a vaccine candidate. The host is protected by stimulating the macrophages to create ROS and NO for the oxidative destruction of intracellular amastigotes (75). O^−2^ and NO prevent *Leishmania* infection. Superoxide is created by the oxidative burst of macrophages in response to phagocytosis during the early stages of *Leishmania* infection (76). In our results, splenocytes from the tuzin-immunized mice group showed a significant increase of NO and intracellular ROS levels when stimulated with SLA of *L. donovani* as compared with the control group, which indicates that tuzin might help eliminate the parasite. The characteristic of active VL in both humans and animals is the impairment of the cell-mediated immune response. Effective vaccine-induced immunity depends on the antigen-specific T-cell response being restored (77). In the present study, we assessed the primary immune response against tuzin by measuring IgG1 and IgG2a antibody levels and quantified the ROS and NO levels in SLA-stimulated splenocytes. While CD4^+^ T cells are the primary source of IFN-γ, it can also originate from other cells like DCs and resident macrophages. Similarly, the regulatory cytokine IL-10 can be produced by B cells, macrophages, DCs, and epithelial cells. However, it is essential to note that we did not investigate specific T subset cell responses and their proliferation, which is crucial to comprehend the exact mechanism underlying the sources of IFN-γ and IL-10 cytokine expression. The results also indicate that Freund’s adjuvants potentially enhanced the protective immune response elicited by tuzin antigens through effector immune cells, providing better protection against *Leishmania* parasites in mice when challenged with wild-type parasites. Freund’s complete and incomplete adjuvants are well-established adjuvants, which are being used in animal studies to determine the antibody response against vaccinated antigens. Therefore, we decided to use Freund’s adjuvant first in this study. However, immunization with a wide range of potential adjuvants such as liposome, MPLA, saponin, alum, BCG, and Montanide (ISA 720) may be tested to improve the immune response against tuzin to produce strong and long-lasting protection against *Leishmania* infection (78).

In conclusion, our study suggests that tuzin has a promising efficacy as a vaccine candidate against *Leishmania*, as it can induce a strong humoral immune response, potentially aid in controlling the infection, and contribute to the elimination of the parasite. However, further studies are needed to fully assess and confirm its effectiveness as a vaccine candidate. These findings bridge important gaps in our knowledge and provide a foundation for further research in understanding and combating *Leishmania* infections.

## Data availability statement

The raw data supporting the conclusions of this article will be made available by the authors, without undue reservation.

## Ethics statement

The animal study was approved by the Institutional Animal Ethics Committee (IAEC), University of Hyderabad. The study was conducted in accordance with the local legislation and institutional requirements.

## Author contributions

MD: Investigation, Methodology, Writing – original draft, Data curation, Formal analysis. PS: Methodology, Validation, Writing – review & editing, Data curation, Formal analysis. VM: Methodology, Validation, Writing – review & editing, Data curation. KK: Methodology, Validation, Writing – review & editing, Data curation. AA: Methodology, Validation, Writing – review & editing, Data curation. MN: Data curation, Formal analysis, Software, Visualization, Writing – review & editing. RM: Conceptualization, Data curation, Funding acquisition, Investigation, Methodology, Project administration, Resources, Supervision, Validation, Visualization, Writing – review & editing.
